# In situ measurement of Scots pine needle PRI

**DOI:** 10.1186/s13007-017-0184-4

**Published:** 2017-05-10

**Authors:** Matti Mõttus, Rocío Hernández-Clemente, Viljami Perheentupa, Vincent Markiet

**Affiliations:** 1 Tekniikantie 1, P.O. Box 1000, FI-02044 VTT Espoo, Finland; 2 Department of Geography, Swansea University, Swansea, SA2 8PP UK; 3 Department of Geosciences and Geography, University of Helsinki, PL 68, 00014 Helsinki, Finland

**Keywords:** Photochemical reflectance index, Needle clip, PRI temporal variation, Sample size

## Abstract

**Background:**

The Photochemical Reflectance Index (PRI) calculated from narrow-band spectral reflectance data is a vegetation index which is increasingly used as an indicator of photosynthetic activity. The leaf-level link between the status of photosynthetic apparatus and PRI has been robustly established under controlled light conditions. However, when a whole canopy is measured instantaneously, the PRI signal is heavily modified by vegetation structure and local variations in incident light conditions. To apply PRI for monitoring the photosynthesis of whole canopies under natural conditions, these large-scale measurements need to be validated against simultaneous leaf PRI. Unfortunately, PRI changes dynamically with incident light and has a large natural variation. No generally accepted procedure exists today for determining the PRI of canopy elements in situ.

**Results:**

We present a successful procedure for in situ measurements of needle PRI. We describe, characterize and test an optical measurement protocol and demonstrate its applicability in field conditions. The measurement apparatus consisted of a light source, needle clip, spectroradiometer and a controlling computer. The light level inside the clip was approximately two-thirds of that on sunlit needle surfaces at midday. During each measurement the needle was inserted into the clip for approximately 5 s. We found no near-instantaneous changes (sub-second scale jumps) in PRI during the measurements. The time constants for PRI variation in light to full shade acclimations were approximately 10 s. The procedure was successfully applied to monitor the greening-up of Scots pine trees. We detected both facultative (diurnal) PRI changes of 0.02 (unitless) and constitutive (seasonal) variations of 0.1. In order to reliably detect the facultative PRI change of 0.02, 20 needles need to be sampled from both sunlit and shaded locations.

**Conclusions:**

We established a robust procedure for irradiance-dependent leaf (needle) PRI measurements, facilitating empirical scaling of PRI from leaf (needle) to full canopy level and the application of PRI to monitoring the changes in highly structured vegetation. The measured time constants, and facultative and constitutive PRI variations support the use of an artificial light for in situ PRI measurements at leaf (needle) level.

## Background

The Photochemical Reflectance Index (PRI) [1], originally called the physiological reflectance index [2], was designed to detect light-activated changes in the epoxidation state of the xanthophyll cycle of green leaves [3]. As the epoxidation state changes, so do the leaf optical properties at 531 nm, influencing the value of PRI,

$${\text{PRI}} = \frac{{R\left( {531} \right) - R\left( {570} \right)}}{{R\left( {531} \right) + R\left( {570} \right)}},$$

where *R*(*λ*) is leaf reflectance at the wavelength *λ* given in nanometers. The dependence of *R*(531) on the epoxidation state of the xanthophyll cycle was demonstrated for both the sunflower leaf and canopy levels by Gamon et al. [3] while 570 nm serves as a reference wavelength. At the leaf level, PRI is robustly (yet ambiguously [4]) related to biochemical and photoprotective energy dissipation processes [1, 5]. Later, PRI has been demonstrated to be a complex indicator of carotenoid pool size and status. For example, in temperate and boreal zones, seasonal variation in chlorophyll and carotenoid pool sizes causes a long-term variation in PRI which clearly exceeds that caused by the xanthophyll cycle [4, 6–8]. Due to the central role of carotenoids (including the xanthophylls) in the photoprotection and non-photochemical energy dissipation, PRI enables improved assessment of carbon fluxes in leaves and canopies [9, 10], especially when applied simultaneously with spectroscopic determination of vegetation physiology and biochemistry [11, 12]. Although PRI does not provide as detailed information on the status photosynthetic apparatus as active fluorescence measurement [13], it allows instantaneous inference using an instrument attached on a remote mobile platform. In a recent review, PRI was found to be a good proxy of photosynthetic efficiency at different spatial and temporal scales [14].

The PRI value measured from a distance, e.g. a tower, aircraft or satellite, corresponds to the canopy, not a single leaf [9]. Due to multiple scattering inside the canopy, the contributions from individual elements are mixed nonlinearly, producing strong and complex structural effects in the remotely sensed PRI [15–17]. Further, considerable variation in leaf PRI can be expected due to pigment pool sizes and the activity of the xanthophyll cycle [18–20]. Additionally, variable contributions of blue sky and direct solar radiation of leaf-hitting irradiance create apparent differences between sunlit and shaded leaf PRI even if none exist in nature [21]. Because of this, the empirical links established between canopy PRI and leaf biochemical composition [22] or instantaneous photosynthesis [9, 23] are not robust [6, 16]. For a reliable application of remotely sensed PRI as a proxy for photosynthetic activity, PRI needs to be scaled to the structural level of the leaf. The scaling needs to be validated by in situ measurements of leaf PRI on scales characteristic to remote sensing instruments.

The finest scales used in remote sensing are smaller than 1 m (e.g., airborne measurements). Even at this scale, in situ measurement of PRI is a challenge in tall forest canopies only accessible via towers, cranes or climbing nets. The task is further complicated by the well-known temporal dynamics of PRI. Leaf PRI changes with time are generally divided into two categories [18, 24]: constitutive (also sometimes called sustainable, time scale weeks to months, associated with pigment pools) and facultative (also called reversible, caused by interconversion of xanthophyll cycle pigments). A representative measure of the constitutive component of PRI can be estimated in field relatively easily as several days or even weeks can be used to collect the sample. The facultative PRI variation, on the other hand, depends on the specific light conditions of the leaf and needs to be measured at the location of the leaf simultaneously with the remote sensing data acquisition. Different rates have been reported in literature for facultative PRI changes. Gamon et al. [3] found the strongest correlation between xanthophyll cycle epoxidation state and leaf reflectance during a 10 min period following irradiance change. Ruban et al. [25] estimated the time half-time for reflectance change as 30–45 s, Bilger and Björkman [26] as ca. 1–2 min, and Evain et al. [27] the full fast adaptation to take place “within seconds”. The changes in shaded leaves are faster (a few minutes) and with a smaller amplitude [18, 26] compared with sunlit leaves (10 min or more). Clearly, this makes the validation of remote sensing of the diurnal (facultative) change of needle PRI an exceptional challenge [28]. No such measurements have been described in literature and no measurement protocol exists today, which motivated us to perform this research.

The determination of a reflectance factor of a surface requires quantification of both the (spectral) radiance reflected by the surface as well as the irradiance it is exposed to [21, 29]. With leaves, the reference signal can be measured by placing a reflectance panel at the exact location of the leaf [27, 30]. This method is most accurate but laborious and, for precise measurements, requires extremely adjustable reference panel fixtures. Furthermore, it is not applicable to needleleaf species as needles do not have a flat scattering surface. Instead of reflectance, needle PRI is determined by its spectral scattering which implies consideration of radiation incident on the needle from all directions [21]. In order to avoid the problems with characterizing incident light, leaf (needle) properties are often determined with “leaf clips” which use artificial light [31–34]. Considering the highly dynamic nature of PRI, a robust protocol for in situ needle PRI sampling with a leaf clip should account for rapid variation in the index when the needle is moved into artificial light and as well as the constitutive changes. To our knowledge, only one leaf clip suitable for measurement of individual needles, the Unispec Leaf Clip Mini (PP Systems, Amesbury Massachusetts, USA), is available commercially. This clip has been used already nearly two decades ago by [35] and also recently by [22] in a setup similar to what is described in this short communication. Together with an appropriate light source and spectrometer, the clip allows for a rapid measurement of the optical properties of a leaf (or a single needle) without removing it from the canopy.

Here, we describe, characterize and test an optical in situ measurement protocol for irradiance-dependent leaf (needle) optical properties. We demonstrate the application of the system to monitoring the changes in Scots pine trees during the start of growing season and separate the facultative and constitutive components of PRI change.

## Results

### Needle adaptation to light conditions

**Fig. 1:**
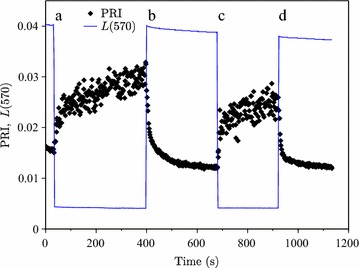
Adaptation of a mat of needles to varying light conditions: PRI and light conditions indicated by the reflected radiance at 570 nm [*L*(570), in W m^−2^ nm^−1^ sr^−1^]. The letters (*a*, *b*, *c*, *d*) identify irradiation transitions plotted in Fig. 2: *a*, *c* are high → low transitions and *b*, *d* low → high transitions

**Fig. 2:**
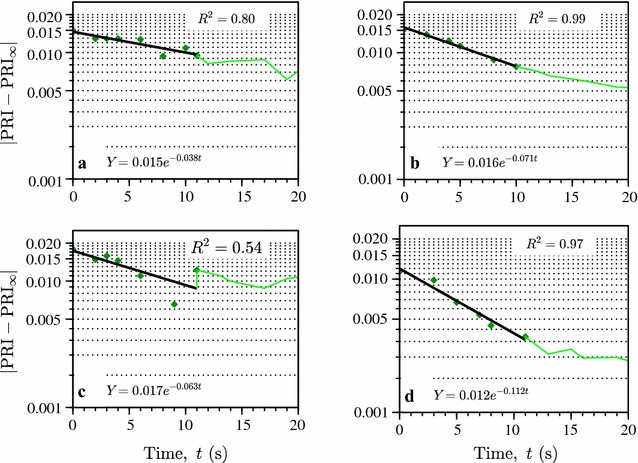
The variation of PRI directly after change in light condition. The results of fitting ${\text{ln Y}} = { \ln }\left| {{\text{PRI}} - {\text{PRI}}_{\infty } } \right|$ for t < 10 s with a linear equation are given in the figure. Subplot letters correspond to the letters in Fig. 1, the* broken line* connects the PRI values measured after the first ten data points used in line fitting

PRI changes remained continuous even when the Photosynthetic Photon Flux Density (PPFD) changed by a factor of ten under controlled laboratory conditions (between the levels of full sunlight and complete shade) (Fig. 1): the first measured PRI value in shade equaled the last one in light, and vice versa. After the change in the light conditions, PRI approached the acclimated value ${\text{PRI}}_{\infty }$ exponentially (Fig. 2) for at least ten first seconds. The time constants of this change calculated from the slope of $\left| {{ \ln }\left( {{\text{PRI}} - {\text{PRI}}_{\infty } } \right)} \right|$ against time were 26.5 and 16.0 s for light to shade transitions (Fig. 2a, c, respectively); for shade to light transitions, the values were 14.1 and 8.9 s (Fig. 2b, d, respectively). More scatter in Fig. 2a and c is explained by the low light (shade) conditions during the measurements. Time constants of the acclimations decreased with time, probably due to incomplete acclimation.

### Field measurements

**Fig. 3:**
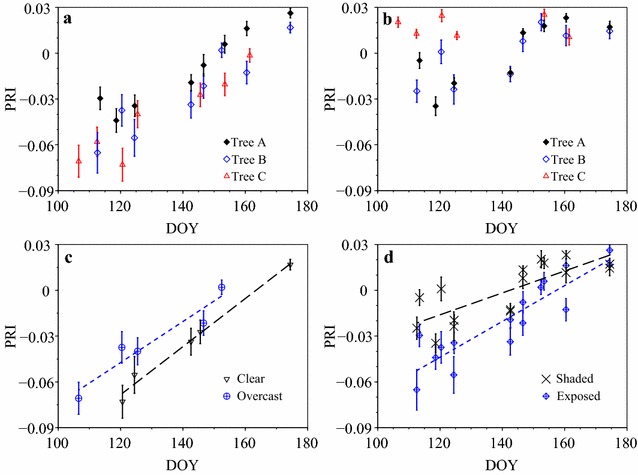
The seasonal dynamics of **a** exposed and **b** shaded shoots; **c** the temporal trajectories of exposed shoots (trees “B” and “C”) on overcast and sunlit days; and **d** the temporal trajectories of sunlit and shaded shoots (trees “A” and “B”) under all sky conditions (overcast, broken and clear). *Bars* indicate 90% confidence limits for mean PRI

The seasonal dynamics of the needles in sun-exposed shoots for three Scots pine (*Pinus sylvestris*) trees measured from beginning of April until end of June (labeled as “A”, ”B” and “C” in Fig. 3a) were similar, although the values for the tree “A” were systematically slightly higher. PRI variation range for the observed period was close to 0.1 with index values increasing monotonously from −0.07 at the beginning of the measurements to close to 0.03 at midsummer. For the shaded shoots, the picture was more complicated: trees “A” and “B” had similar increasing trends while tree “C” had no apparent systematic temporal variation (Fig. 3b). For pooled measurements of sun-exposed shoots of trees “B” and “C” (excluding tree “A” for clarity because of its systematically higher values), PRI on overcast (diffuse conditions) days was by 0.02 higher compared with clear skies throughout the season (Fig. 3c). We excluded measurements under broken skies for clarity. The seasonal trajectories of PRI values for the two trees which have similar trends (“A” and “B”) converged at 0.02–0.03 around midsummer [Day of Year (DOY) 180, Fig. 3d]. The same is evident for the sun-exposed shoots of tree “C” (Fig. 3a): the PRI of shaded shoots of tree “C” was 0.02 regardless of DOY (Fig. 3b).

The standard deviation of the PRI values for the 20 needles measured in a single shoot varied between 0.01 and 0.08 with a median of 0.025. When the measurements of the four shoots corresponding to one canopy location of one tree were combined (80 measurements), the median PRI standard deviation increased to 0.031 while the range remained essentially the same.

## Discussion

Our results are generally in line with what has been reported before. The most variable results have been reported for the time constant of PRI change. Our test indicated no instantaneous (sub-second scale) jump in PRI at light conditions change, which agrees with many—but not all—earlier investigations. Near-instantaneous changes when a needle is inserted into the clip would have made it impossible to use artificial light for PRI reference measurements. Also, the ranges in constitutional and facultative PRI variations agree well with what has been reported for Scots pine and green leaves in general [8, 22, 36]. This supports the use of needle clips in PRI monitoring.

The time constant values reported here were measured to get indication on the time window available for in situ needle measurements. The time constants obtained by us were similar to those reported by Ruban et al. [25] although we also measured a value below 10 s. The determination of time constants, however, is not a straightforward task as PRI change is not caused by a single mechanism and exponential change (an assumption required by the definition of the time constant) is only an approximation. In literature, several mechanisms for PRI change have been reported working on different time scales [37]. Trans-thylakoid pH gradient controls dynamic thermal energy dissipation, triggering de-epoxidation of the xanthophyll cycle pigment violaxanthin to zeaxanthin under excess light [8, 25]. The dissipation can become sustained [8, 37, 38], i.e., independent from instantaneous PPFD. The different mechanisms can likely be behind the longer PRI variation accompanying the dissipation of excess radiation that cannot be processed through the photosynthesis. Activation of these longer-term variations affect the retrieved PRI time constant. Also, according to the definition of a time constant, its value depends on the amplitude of PRI variation. The PRI range (0.012, 0.025) used in the time constant calculations was smaller than expected for full adaptation [18]. Nevertheless, it is clear that to be sure that in situ PRI values are comparable with remote sensing data, the measurement cannot take more than a few seconds. While slower measurements may detect a difference between exposed and shaded needles, the facultative component in the PRI variation would be strongly suppressed.

In laboratory, we induced a 10-time change in incident PPFD. This is similar to the natural extremes for shaded and sunlit conditions, but much larger than what is expected for a needle entered into the clip: the irradiation in the clip is between the values in the sun and in the shade. Although this need not increase the time constant, it affects the acclimated PRI value, ${\text{PRI}}_{\infty } ,$ and decreases the amplitude in PRI variation. Unfortunately, we were not able to measure the temporal dynamics of needle PRI in the clip. As the clip was held manually, the shaking caused small movements of the needle and, possibly, leaking of sunlight into the clip. However, even for apparently error-free clip PRI time series, we could only see slow (time scale: tens of seconds to minutes) changes in PRI, possibly due to the instability of the spectrometer. No monotonic rapid PRI variations were recorded for the first 10 s (data not shown). Therefore, we expect our measurements to truly represent in situ values of needle PRI.

The sunny and cloudy measurements in Fig. 3c are systematically different throughout the measurement period. The difference between the two quasiparallel lines equals facultative PRI difference caused by instantaneous light conditions. Clearly, this facultative difference was considerably smaller than the seasonal constitutive PRI change quantified by overall variation in the index with DOY. PRI was dominated by needle pigment pool sizes associated with seasonal development and canopy position [18, 39] driven by the ratio of carotenoids to chlorophyll [4, 6, 8, 40]. For the beginning of the measurement period, the recorded PRI variation caused by light (difference between the two lines in Fig. 3c) was approximately the same as for exposed-shaded needle difference (Fig. 3d) as suggested by Gamon and Berry [18]. The seasonal range of PRI matched that measured in Hyytiälä [8] and the facultative range was within that suggested by Gamon and Bond [31]. However, some differences between the trees existed. The shaded shoots of tree “C” showed little seasonal variation (Fig. 3b) and were excluded from Fig. 3d. A reason could be that the shaded shoots of “C” were more shaded than those of the other two trees (data not shown). The somewhat higher PRI values of “A” compared with “B” and “C” were likely caused by natural variation.

The biggest surprise was the convergence of the two lines in Fig. 3d. Apparently, a difference between the PRI of sun-exposed and shaded needles is not trivial in midsummer. The measured shaded shoots were selected to be as shaded as possible within the reach of instrument operator at the bottom of the canopy. As Scots pines are shade-intolerant, the shaded shoots were sparse and short, easily distinguishable from the sun-exposed shoots used in the study. It may be possible that in midsummer, they still receive sufficient radiation to have pigment pool ratios similar to sun-exposed needles.

The standard deviation of the measured needle PRI was of the same magnitude as the difference between shaded and exposed needles, or the PRI change in exposed needles due to light conditions. Numerous sources of PRI variation exist. Natural PRI variation, uncertainties in measurement (instrument noise and stability) and sampling effects (the location of the needle inside the clip) cannot be separated in the data. Our results thus suggest that a large sample (tens of needles) is required to reliably detect temporal and spatial PRI variations. For example, if we assume that the measured needle PRI standard deviation of 0.03 represents all variation, and the required standard error of the mean PRI is one-third of the exposed—shaded needle PRI difference of 0.02, 20 needles need to be measured. The measured needles should not belong to one shoot as the number of measured shoots per sample (one or four) had an effect (although not a large one) on PRI variation. A measurement of 20 needles in one shoot using the methods described here takes approximately 5 min. Within 20 min of a remote sensing acquisition, 160 needles can be sampled to determine the PRI of both shaded and exposed needles.

## Conclusions

Despite the numerous complications associated with PRI field measurement, adequate and reliable sampling of PRI for remote sensing reference measurements can be achieved with artificial light for a whole canopy under natural conditions if the measurements are performed rapidly (within 5 s) on a sufficient sample (at least 20 needles from different shoots). For the test species, constitutive changes dominated in PRI temporal variation on monthly to seasonal scales and also in within-canopy PRI variation. At the beginning of peak growing season, however, constitutive variation within the measured treed was small and facultative changes were the main cause for PRI variation with light conditions. With proper upscaling, these measurement protocol has significant potential application to validate the connection between remotely measured canopy PRI and the biochemical status of the foliage visible to the sensor, allowing to build a robust link between remote sensing and vegetation photosynthetic activity.

## Methods

### Time constants for needle adaptation

To determine the speed of variation in needle optical properties with incident PPFD, a flattened mat of Scots pine needles still connected to a watered shoot was exposed to abruptly changing light conditions in laboratory. Variations in PPFD were caused by quickly (within 100 ms) inserting and removing a neutral density filter. The unfiltered PPFD equaled 1000 μmol/m^2^/s, the filter reduced PPFD by 89%. After the needle mat had been illuminated for 5 min, it underwent two cycles of adaptation to reduced and full light: two rapid decreases in PPFD (denoted by letters “a” and “c” in Fig. 2) and two increases (“b” and “d”). The radiance reflected by the needle mat was recorded by ASD Handheld VNIR spectroradiometer set to log at 1 s intervals looking at the sample at approximately 45 angle from a distance of 4 cm. The spectral resolution of the spectrometer was approximately 3 nm. As a reference, WS-1 diffuse reflectance standard (Ocean Optics, reflecting material Spectralon, reflectance >99%) was measured before and after the needle measurements. In addition to PRI, we monitored reflected radiance at 570 nm as an indicator of incident PPFD.

The time constant *τ* is defined for an exponential process *f*(*t*) = *ke* ^−*t*/*τ*^ which approaches zero as *τ* → ∞. Therefore, we modeled PRI as an exponentially decreasing function of time,

$$\left| {{\text{PRI}} - {\text{PRI}}_{\infty } } \right| = ke^{ - at} ,$$

where ${\text{PRI}}_{\infty }$ is the asymptotic PRI value under current light conditions estimated from measurements as 0.025 and 0.012 for dark and bright illumination conditions, respectively. After regressing ${ \ln }\left| {{\text{PRI}} - {\text{PRI}}_{\infty } } \right|$ linearly against time for the first 10 s after irradiance change, we determined the time constant of PRI transition as *τ* = 1/*a*.

### Measurement system

The measurement system was built around the PP Systems (Amesbury, Massachusetts, USA) UNI501 Mini Leaf Clip suitable for both needle and leaf measurements, and the PP Systems UNI410 bifurcated optical cable. Low OH optical cables were used to direct light from the Ocean Optics (Dunedin, Florida, USA) HL-2000 tungsten halogen light source (power 5 W) through Ocean Optics FHS-UV in-line filter holder into one end of the bifurcated cable. The other end of the bifurcated cable was connected to an Ocean Optics USB4000-VIS–NIR portable modular spectrometer.

During measurements, the sample (needle) was partly inserted into the needle clip. To facilitate needle measurements, the clip has groove opening at the center of the blunt end of the clip. Light was directed to the sample at a 30° angle from its normal using a bifurcated optical cable consisting of a bundle of 50 micron fibers. The total diameter of the bundle was 0.86 mm. According to the manufacturer, the distance from leaf surface to end of the fiber, measured along the optical axis, was 1.8 mm. Because of the groove, a needle would be located somewhat closer to the end of the fiber bundle.

The nominal input voltage of the tungsten lamp was 12 V provided in field by 10 NiMH cells. The voltage was monitored by a small digital voltmeter attached to the lamp. According to laboratory tests, lamp optical output was insensitive to voltage variations and remained stable even if the voltage dropped below 9 V. Before entering the bifurcated cable, the light passed a filter holder with an Edmund Optics 785 nm OD 6 blocking notch filter. The filter completely removed light between 775 and 805 nm; in other parts of the spectrum used in the study the transmittance was above 90%. The filter can be used to track radiance emitted as fluorescence close to the widely used O_2_-A absorption band while retaining most of the reflectance spectrum. The filter holder was covered with a black-walled cardboard box allowing no light to enter from outside.

The light reflected or emitted from the sample was measured with the spectrometer powered and controlled by a notebook computer running the OceanView (Ocean Optics, Dunedin, Florida, USA) spectroscopy application. The spectral resolution of the spectrometer was 1.5–2.3 nm (full width half maximum). A total of 3648 spectral channels were recorded during each scan, thus heavily oversampling in the spectral domain. The useable range of the system was approximately 400–900 nm, limited by lamp power at the short and spectrometer sensitivity at the long wavelengths. The measured spectra were smoothed with a Savitzky-Golay filter (order = 2, *N* = 15).

The system (bifurcated optical cable connected to the spectrometer) was repeatedly calibrated in laboratory by comparison with factory-calibrated ASD Handheld VNIR (Analytical Spectral Devices, Boulder, Colorado, USA) and SVC HR1024i (Spectra Vista Corporation, Poughkeepsie, New York, USA) spectroradiometers. The calibrations of both spectrometers were traceable to NIST with absolute accuracy of approximately 5%. The accuracy and stability of the needle measurement system was, however, considerably below this level because of repeated assembly and disassembly of the system, (re)connecting the SMA 905-teminated optical cables and cleaning of connector surfaces.

The photosynthetic photon flux density (PPFD) received by the target was determined using the Spectralon measurements performed in field. The approximate value was 600–700 μmol/m^2^/s, which constitutes approximately two-thirds of that on sunlit needle surfaces in Finland in midsummer. The actual PPFD varied for the reasons mentioned above in relation calibration stability, but also because of the variations in the distance between fiber tip and target. The beam exiting the fiber was conical with approximately 25° divergence. The variation of the distance between fiber clip and the needle surface by 0.5 mm, e.g., due to the location of the needle in the groove, would lead to approximately 30% variation in surface irradiance.

### Field measurements

Spectral measurements were performed on three different Scots pine (*Pinus sylvestris*) trees in Kumpula, Helsinki, Southern Finland (60°12′N, 24°57′E). The naturally growing trees were chosen for their accessibility: they were growing partly in the open with foliage accessible from the ground. The measurements were taken throughout the spring and early summer (between 04 April 2015 and 23 June 2015) to cover a wide range of illumination and environmental conditions and physiological status.

Needle optical properties were measured separately for the sunlit and shaded shoots of the trees. We selected four shoots receiving direct sunlight for a large part of the day (including noon) from each tree. Correspondingly, we chose four shoots which were always in (partial) shade. Visually, the sun-exposed shoots were easily distinguished from shaded ones as the latter were much sparser and shorter. The shoots were marked with ribbons and the same shoots were always used for measurements. The illumination conditions were validated by taking a leveled hemispherical photograph at the location of each shoot and calculating the light regime with WSL Hemisfer (Birmensdorf, Switzerland) software. For each shoot, 20 needles were measured, resulting in 80 needles measured for each tree and each canopy location. Only last-year needles were measured.

Before starting the measurements, the halogen light source and the spectrometer were turned on for at least 10 min. The integration time of the spectrometer, approximately 10 ms, was set based on white Spectralon measurements. A single needle was inserted along the small groove of the clip. The closed clip was covered with a visor made of black adhesive tape to limit the amount of stray light. 20 scans were averaged in software to constitute a needle measurement. The time a needle was kept in the clip was approximately 2–3 s before the reflectance signal was recorded. The Spectralon panel was measured after each 5 needles. Empty needle clip was regularly measured for monitoring the cleanness of the optical pathway. In case there appeared to be distractions, e.g. resin, the pathway was cleaned using cleaning alcohol, a narrow brush and compressed air. Two people were required for measurement, one to insert the needle and the other to operate the computer. The measurement of 4 shoots took approximately 20 min.

The measurements were preferably performed under uniform sky conditions (clear sky or fully overcast). However, due to meteorological characteristics of the study area, measurements also had to be taken under broken skies. In this case, we waited for 10 min after a cloud shadow had passed before measuring. Detailed field logs including weather conditions and time of measurements were composed for each measurement day. A total of 3566 individual needle PRI values were recorded during the campaign.
